# Development of monitoring and evaluation guidelines for Indonesia’s national Early Warning Alert and Response System: aiming for a standardized and systematic assessment

**DOI:** 10.5365/wpsar.2026.17.2.1320

**Published:** 2026-04-20

**Authors:** Shingo Nishiki, Irma Gusmi Ratih, Nur Assyifa Daiyah Fillah, Rizki Dinar Winiar, Isamu Kuboki, Rendy Manuhutu, Lia Septiana, Eka Muhiriyah, Triya Novita Dinihari

**Affiliations:** a Project for Strengthening Capacity for Early Warning and Response to Infectious Diseases, Japan International Cooperation Agency, Jakarta, Indonesia.; b Directorate of Surveillance and Health Quarantine, Ministry of Health, Jakarta, Indonesia.; * These authors contributed equally to this work as shared first authors.

Monitoring and evaluation (M&E) are essential for establishing and maintaining effective communicable disease surveillance and response systems, as well as for enhancing capacity. (*1*) Recent public health emergencies, particularly the COVID-19 pandemic, have underscored the importance of these advancements globally. In Indonesia, the Early Warning Alert and Response System (EWARS) was implemented in 2009 as a web-based syndromic surveillance platform for infectious diseases. (*2*) Like many other countries, Indonesia has faced challenges related to emerging and re-emerging infectious disease outbreaks. (*3*, *4*) These experiences have exposed gaps in key surveillance areas, such as outbreak detection, laboratory diagnostics and control measure implementation, highlighting the need to strengthen national capacity. (*3*-*5*) Moreover, the 2023 World Health Organization (WHO) *Joint external evaluation of the International Health Regulations (2005) core capacities of Indonesia* encouraged the Government to regularly assess its surveillance system to identify priority gaps and implement targeted interventions. (*6*)

In response, the Indonesian Ministry of Health (health ministry) initiated efforts in 2024 to integrate EWARS into a standardized systematic M&E scheme for its national infectious disease surveillance system in collaboration with the WHO Representative Office for Indonesia and the Japan International Cooperation Agency (JICA). This initiative aims to translate international standards into specific and practical operations for Indonesia, addressing local needs and contexts. This brief report outlines these efforts and offers insights for future development.

Indonesia’s EWARS operates across four levels: national, provincial, district and subdistrict. (*2*, *7*) At the subdistrict level, primary health-care centres serve as the main reporting units. The system monitors 24 diseases and syndromes through indicator-based surveillance (IBS). These diseases include acute diarrhoea; acute flaccid paralysis; acute jaundice syndrome; acute respiratory infections; animal bites; anthrax; avian influenza in humans; bloody diarrhoea/dysentery; chikungunya; cholera; COVID-19; dengue; diphtheria; hand-foot-mouth disease; influenza-like illness; leptospirosis; malaria; measles; meningitis/encephalitis; neonatal tetanus; non-neonatal tetanus; pertussis; pneumonia; and typhoid fever. (*2*, *8*) The system provides weekly reports, and also incorporates event-based surveillance (EBS), which requires reporting of 20 disease events within 24 hours of detection. (*2*) Data are primarily transmitted from reporting units to the national server, which contains aggregated IBS case counts and individual EBS reports. (*2*, *9*) When the IBS system detects an unusual pattern exceeding a predefined alert threshold, alerts are automatically generated for relevant agencies, prompting primary response activities. (*2*, *9*) Responses to EBS notifications are promptly triggered upon detection.

The M&E for EWARS has primarily focused on performance indicators such as report submission completeness and timeliness, the number of alerts and response rate. (*2*) Regular stakeholder meetings have been held at various levels to review and discuss these indicators. However, there remain concerns that these indicators do not always ensure the quality of surveillance, highlighting the need for new tools that enable systematic assessments and in-depth analysis beyond the scope of existing indicators.

The developed EWARS M&E scheme adopts an external cascade evaluation approach, whereby national authorities evaluate provincial activities, provinces assess districts, and districts review reporting units. This approach can ensure the generality, reproducibility and granularity of the assessment compared to self-assessment methods. (*10*) Accordingly, three tailored guidelines were developed, aligned with the specific roles of each evaluation target. These guidelines comprise four key components: 1) assessment sheets; 2) assessment summary tables; 3) user manuals; and 4) preparation checklists. The assessment sheets include approximately 100–130 questions, comprising both closed- and open-ended questions, to evaluate the quality of EWARS qualitatively and quantitatively across eight domains (**Box 1**). These domains include facility equipment and environment, performance indicators, IBS, EBS, laboratory testing, data management, response activities, and information-sharing and communication, which are consistent with the WHO and EWARS guidelines. (*1*, *2*) Each domain consists of several topics, each accompanied by multiple questions. In most cases, closed-ended questions with single- or multiple-choice answers are followed by open-ended questions that allow respondents to provide additional details, particularly in cases where they encounter challenges. The summary tables compile results from key closed-ended questions on a two- or three-point scale, alongside evaluator recommendations. The manuals assist interviewers and data-entry operators in accurately interpreting questions and formulating appropriate responses. The checklists specify the documents and data that respondents should prepare in advance. Additionally, widely accessible free online tools are used to streamline data management processes, including data collection and visualization. During evaluations, data are entered into online forms in real time, with the results instantly visible on dashboards in various formats immediately after data entry.

**Table:** Box 1. Eight evaluation domains for Indonesia’s national Early Warning Alert and Response System

No.	Evaluation domain
1	Facility equipment and environment
2	Surveillance performance indicators
3	Indicator-based surveillance
4	Event-based surveillance
5	Laboratory testing and records
6	Data management and analysis
7	Response activities
8	Information-sharing and communication

The first trial was conducted in East Kalimantan Province in February 2025, involving site visits to provincial and district health offices and primary health-care centres to assess effectiveness and identify areas for improvement. Prior to fieldwork, stakeholders received online orientation on assessment procedures. During onsite evaluations, evaluators conducted interviews, entered data and prepared assessment summaries with support from health ministry supervisors. Although each of the site visits initially lasted approximately 1–2 hours, the duration decreased with successive rounds. During the trial, online feedback questionnaires were distributed to the participants, and wrap-up workshops were held to facilitate the exchange of ideas between evaluators and respondents. This trial demonstrated that the M&E guidelines and tools functioned effectively, highlighting performance variations between facilities and identifying key challenges. Participants rated the guidelines as highly practical, but suggested modifications to questions for the sake of clarity and requested more time for preparation.

Following the trial, questions that caused interpretative or response challenges were extensively revised, and the user manuals and preparation checklists were refined accordingly. For instance, terms that could be interpreted differently by respondents, such as the requirements relating to training or to the conduct of analyses, necessitated the specification of criteria along with typical examples. To minimize any miscalculations in questions requiring numerical responses, such as proportions, a formula was added to the questionnaire. Additionally, the quality of the pre-prepared data set was standardized, detailed breakdowns of the submitted data were specified, and target periods were clarified.

Using these updated materials, the second trial was conducted in Banten Province in May 2025. Based on lessons learned, a more precise pre-trial briefing was provided to subnational authorities, with extended preparation time. As a result, the second trial demonstrated shorter assessment times and improved guideline usability, indicating increased operational efficiency and greater feasibility of EWARS M&E activities. The guidelines were subsequently published with the necessary revisions in July 2025. (*11*)

Overall, these trials were deemed successful, providing valuable insights for future implementation. First, the summary table offered multiple benefits, as it can be quickly generated by simply clicking checkboxes, with results calculated automatically. It serves as a convenient reference for review, facilitates timely feedback and functions as an evaluation certificate. Second, the effective use of online tools addresses constraints related to time, budget and human resources. Third, the use of free online tools supported efficient data collection and visualization. Additionally, dashboards facilitated data-driven communication among stakeholders, which is expected to enhance data-informed decision-making. (*12*) Also, periodic assessments with quantitative indicators derived from key questions are expected to enable the monitoring of progress in surveillance capacity over time. Furthermore, trial participants recommended conducting online M&E activities in parallel with in-person assessments in future implementations.

Finally, the trials underscored that evaluation is not merely about assessment but also about learning. Interactive exchanges fostered knowledge-sharing, benefiting both evaluators and respondents. Evaluators gained clearer insights into existing challenges and effective practices in field surveillance, while respondents enhanced their understanding of standard procedures. Highlighting these mutually beneficial outcomes and best practices can promote greater stakeholder engagement in M&E activities and drive improvements in surveillance quality. Moreover, these findings suggest that similar approaches can be tailored to address the specific surveillance challenges faced in various low- and middle-income country settings, while promoting collaborative relationships and cultivating a culture of continuous improvement across different health-care sectors.

In conclusion, the initiative to establish a systematic M&E framework for EWARS in Indonesia began in 2024 with the development of guidelines and online tools through pilot activities. Although still in its early stages, this effort represents a significant step towards regular, large-scale implementation. Ultimately, this initiative has the potential to strengthen Indonesia’s surveillance system and contribute to capacity-building in other countries by offering practical insights into structuring surveillance M&E systems.
